# Linguistic Diversity and Traffic Accidents: Lessons from Statistical Studies of Cultural Traits

**DOI:** 10.1371/journal.pone.0070902

**Published:** 2013-08-14

**Authors:** Seán Roberts, James Winters

**Affiliations:** 1 Seán Roberts Max Plank Institute for Psycholinguistics, Nijmegen, The Netherlands; 2 James Winters Language Evolution and Computation Research Unit, School of Philosophy, Psychology and Language Sciences, The University of Edinburgh, Edinburgh, United Kingdom; Queen's University Belfast, United Kingdom

## Abstract

The recent proliferation of digital databases of cultural and linguistic data, together with new statistical techniques becoming available has lead to a rise in so-called *nomothetic* studies [1]–[8]. These seek relationships between demographic variables and cultural traits from large, cross-cultural datasets. The insights from these studies are important for understanding how cultural traits evolve. While these studies are fascinating and are good at generating testable hypotheses, they may underestimate the probability of finding spurious correlations between cultural traits. Here we show that this kind of approach can find links between such unlikely cultural traits as traffic accidents, levels of extra-martial sex, political collectivism and linguistic diversity. This suggests that spurious correlations, due to historical descent, geographic diffusion or increased noise-to-signal ratios in large datasets, are much more likely than some studies admit. We suggest some criteria for the evaluation of nomothetic studies and some practical solutions to the problems. Since some of these studies are receiving media attention without a widespread understanding of the complexities of the issue, there is a risk that poorly controlled studies could affect policy. We hope to contribute towards a general skepticism for correlational studies by demonstrating the ease of finding apparently rigorous correlations between cultural traits. Despite this, we see well-controlled nomothetic studies as useful tools for the development of theories.

## Introduction

Recent studies have been uncovering some surprising links between cultural traits. For example, between chocolate consumption and the number of Nobel laureates a country produces [9], between the number of phonemes in a language and distance from East Africa [3], between a language's tense system and the propensity to save money [2], between the quality of the sounds of a language with the amount of extra-marital sex [6] and genetic influences on political outlooks [4], [5].

Nomothetic studies (statistical analyses of large-scale, cross-cultural data) are possible because of recently available, large-scale databases and new statistical techniques which give social scientists more statistical power to explore the relationships between cultural phenomena. They are quick and easy to perform. However, there are several potential problems with this type of study. While it is common knowledge that correlation does not imply causation, there are few studies that utilise methods to address the problems caused by cultures being related by descent (Galton's problem, [10], see [11]) and by geographic diffusion [12]. Furthermore, the data used in these studies is inherently coarse, which can create apparent correlations. There is also the problem of inverse sample-size: with larger amounts of data, a spurious correlation becomes more likely.

These problems combine to increase the likelihood of finding correlations between cultural traits. In this paper we demonstrate that it is possible to link a wide variety of cultural traits in a chain of correlations, all of which may seem rigorous, but some of which are not plausibly causal. In fact it may be possible to find apparently rigorous evidence for any hypothesis. It is also tempting to fit post-hoc hypotheses to correlations that fall out of nomothetic studies. However, without a proper awareness of the problems, this kind of study could be damaging to the direction of research and public policy.

The inter-connectedness of cultural traits that we demonstrate raises problems for the usefulness of statistical analyses as independent sources of knowledge. However, we suggest that nomothetic studies should be seen as hypothesis-generating tools that can work with and direct other methods such as idiographic studies, computational modelling, experiments and theoretical work [13], [14]. We also suggest some methods that might improve statistical inference and insight in nomothetic studies, including phylogenetic techniques and inferred causal graphs [15]. To our knowledge, this is the first application of high-dimensional causal graph inference to cultural and linguistic data.

The paper is ogranised in the following way. First, we summarise some nomothetic case-studies. We outline some problems facing nomothetic studies and suggests some criteria for evaluating them. Our results section demonstrates a chain of statistically significant links between cultural traits, followed by a short discussion. Finally, we suggests some solutions to the problems discussed.

## Nomothetic Studies

One example of a nomothetic study used the World Atlas of Language Structures (WALS) [16] database to demonstrate that a community's size is related to the morphological complexity of its language [1]. This is a well controlled statistical test which is robust across language families. The suggested mechanism behind this link, motivated by prior theory (e.g. [17]–[19]), is the difference between adult and child language learning. Because larger communities are more likely to have more adult second language learners, and adults are worse at learning morphology than children [20], this puts pressure on languages of large communities to become less morphologically complex over time.

Another study discovered a link between areas with a prevalence of a recently mutated geneotype and populations with tonal languages (languages where lexical contrasts can be made by altering pitch patterns) [7]. This generated the hypothesis that linguistic structure could be affected by small genetic biases over time. Because the baseline level of chance correlation is difficult to estimate, the statistical significance was computed by comparing the strength of the link to the strength of the link between thousands of other linguistic and genetic variables. By demonstrating that the hypothesised link was stronger than competing hypotheses, a convincing claim was made for the further experimental investigation of this hypothesis. In order to develop the basis of the general theory, a follow-up experimental study found support for part of the hypothesis in that there are individual differences in the perception of pitch [21], and a computer simulation demonstrated that such differences could influence linguistic structure in the long-term [22].

A number of studies have demonstrated links between a community's size and the number of contrasting sounds (phonemes) in its language. Hay & Bauer demonstrate a positive correlation between population size and phoneme inventory size [8] (replicated in [3], [23]). However, recent analyses using larger samples and accounting for the relatedness of languages find no such correlation [24], [25]. While the original results might be debatable, and despite the proposed link between phoneme inventories and social structure being well-established (e.g. [26]), the debate surrounding the original nomothetic study did offer the opportunity for the development and application of a wide variety of statistical techniques. This includes the use mixed effects models that can control for nested data by placing predictors at differing levels [24], [27].

Ecological aspects have also been shown to predict linguistic variables. Correlations are reported in [28] between the average sonority of a language – the average amplitude of its phonemes – and the local climate and ecology. The proposed hypothesis includes people in warmer climates spending more time outdoors, and sonorous sounds being more effective at communicating at a distance. This finding was extended to account for cultural features such as the amount of baby-holding, levels of literacy and attitudes towards sexual promiscuity [6]. The link with sexual attitudes is hypothesised as being due to sexual inhibition discouraging speaking with a wide open mouth. Below, we show that population size also correlates with these variables.

Nomothetic studies can also straddle relatively disparate fields. For example, two studies find a correlation between the distribution of political attitudes (individualist versus collectivist) and the prevalence of a gene involved in the central neurotransmitter system 5-HTTLPR [4], [5]. The social sensitivity hypothesis suggests that, because alleles of this gene affect the likelihood of a depressive episode under stress [29], communities with a higher prevalence of this gene will require more social support. Therefore, these communities will develop to be more collectivist rather than individualist. However, a missing element of this hypothesis is how a difference in the distribution of these alleles emerged in the first place. By exploring the inter-connectedness of many different variables, we develop a hypothesis which suggests that migration and environmental conditions could bring about this distribution (see the section ‘Causal graphs’).

### Media attention

Nomothetic studies often demonstrate surprising links between cultural phenomena. For this reason, they often receive media attention. For example, based on research that flavenoids found in chocolate benefit cognitive function [30], a study demonstrated that countries which have a higher per-capita consumption of chocolate produce more Nobel laureates [9]. The study used a simple linear correlation, without controlling for any other factors, yet received a large amount of media attention [31]–[33]. Even though the study may have intended the correlation to be interpreted as an example of spuriousness, it failed to control for other factors and possible confounds. This is an example of a misapplication of statistical techniques. While this particular study may seem harmless, below we use the same data to demonstrate correlations which appear to have more serious implications for public attitudes and policy.

Another study that has received media attention is the finding that speaking a language that has an overt morphological future tense predicts economic behaviour such as the propensity to save money [2]. This study was discussed before publication in public forums online [34]–[36] and in the media [37]–[41]. The media typically exaggerate the implications of this type of finding and try to link it to current events rather than emphasise the long-term change implied in most studies. For instance, one popular science review of study [2] suggested “Want to end the various global debt crises? Try abandoning English, Greek, and Italian in favor of German, Finnish, and Korean.” [38].

## Problems

In this section we review three problems that cause spurious correlations in nomothetic analyses of cultural phenomena.

### Galton's problem

One of the better-known issues facing nomothetic researchers is that of *Galton*'*s Problem* [10]. Named after Sir Francis Galton, following his observation that similarities between cultures are also the product of borrowing and common descent, Galton's Problem highlights that researchers must control for diffusional and historical associations so as to not inflate the degrees of freedom in a sample [42].For example, the likely magnitude of a correlation emerging between two independent traits is much higher if the traits diffuse geographically than if they change randomly [34]. Cultural traits, then, form a complex adaptive system [43] where some links are causal and some links are accidents of descent. For this reason, we would expect to see spurious correlations appearing between unlikely cultural variables.

Ascertaining the degree of independence between cases is a concern that has a long history in cross-cultural research [44]. Numerous methods have been proffered as potential solutions, notably: spatial autocorrelation, phylogenetics and generalised linear mixed models [12]. One debated difference is the amount of horizontal transmission that occurs in cultural traits [45]–[48]). While there are well-developed models for genetic evolutionary change that are used in phylogenetic analyses [49], it is less clear whether they are suitable for assessing cultural change. Complicating this is the difficulty of identifying cultural traits in the past due to a lack of comparative evidence and the transience of cultural traits such as spoken language.

Large datasets and complex relationships are dealt with regularly in fields like genetics. However, there is an active debate about the role of statistics in causal inference [50]. Neuroscience studies involving brain imaging also deal with large, complex datasets. However, spurious correlations are also a problem here [51], [52], and the inference based on some advanced techniques have been recently questioned [53]. Despite an awareness of the problem, there are few studies with a sophisticated approach to addressing it. In general, review of statistics used in studies of culture and language may be less rigorous than in other fields [54]. This might suggest that, for researchers, the crux of the problem is a lack of tools, not a lack of awareness of the problem.

### Distance from data: Are linguists the main drivers of changes in consonant inventory sizes?

Nomothetic studies often use databases that exhibit a distance from the real data. This is particularly salient when the datasets consist of statistically rare observations i.e. one researcher generated all the data for one particular data point. The amount of variance and selection bias introduced via the process of getting from actual data-collection to the database in question can be problematic in terms of analysis.

An illustrative example is found in classifying the size of a particular constant inventory for WALS. WALS determines its consonant inventory size data by binning raw consonant counts into the following categories: small (6–14), moderately small (15–18), average (22±3), moderately large (26–33) and large (34 or more) [55]. These categorical variables are useful for the context in which WALS was created: to highlight the geographic distribution of typological diversity around the globe. However, such coarse lumping into categorical variables might inflate error, especially when the variables could lend more weight to finding a particular correlation than another.

Still, there can also be considerable distance between the observations of different field linguists. Take the reports of consonant inventory sizes for the Wichí language – a member of the Matacoan language family spoken in various parts of South America's Chaco region [56]. For instance, in 1981 when Antonio Tovar published an article on the Wichí's phoneme inventory [57], he arrived at a figure of 22 consonants. Jump forward 13-years to 1994 and Kenneth Claesson's report [58] would tell you the Wichí are down to just 16 consonants. This is just one of what is likely be many examples of huge degrees of variation in linguistic observations for rare languages. The difference in reports would be enough to change the categorical value in WALS from an average consonant inventory size to a moderately small one.

There are several explanations for the variance in such reports. Some instances could be genuine differences between speech communities in the form of dialectal variation. Other reasons take the form of theoretical motivation. Claesson, for instance, chose to omit glottalized consonants from his description of Wichí. His rationale being that these “are actually consonant clusters of a stop followed by a glottal stop” [56], [37]–[38]. In summary, both sources of data are sensitive to the biases of the researchers: for each language, or dialect, these observations are reliant on the choices of potentially one researcher, at a very specific point in time, and with only a finite amount of resources. We believe such sources of variance are not limited to phoneme inventory data, but rather are endemic in these sorts of data, which leads to the problem of having “too many variables (but too little data per variable)” [59].

### Inverse sample size problem

Whilst we believe big data is a valuable resource for social scientists, the type of big data collected, as well as the types of questions asked in relation to these datasets, are of a fundamentally different nature to those found in other areas that rely on large datasets. Pick up any statistical textbook and it is likely you will read something along the lines of “as is intuitively obvious, increases in sample size increase statistical power” [60]. This is certainly true on an absolute basis where there is a decrease in the noise-to-signal ratio. For instance, the extremely small sample sizes in neuroscience are probably responsible for the overestimates of effect size and low reproducibility of results ([52]; but also see [61] for a more general discussion on this problem across all sciences). We have also seen great successes in physics where large amounts of data were crucial in the discovery of the Higgs Boson [62], [63] or in astronomy with the spectroscopic survey of millions of stars (the Sloan digital sky survey [64]). Yet, as Gary Marcus recently noted, large datasets in physics are characterised by certain properties:

“Big Data can be especially helpful in systems that are consistent over time, with straightforward and well-characterised properties, little unpredictable variation, and relatively little underlying complexity” [65].

It is tempting to apply the same principled reasoning to the nomothetic approach to culture. However, nomothetic studies tend to rely on data that falls on the opposite end of the spectrum: these datasets tend to be incomplete, complex and based on inconsistent criteria. Problems such as those in the case of the Wichí's consonant inventory are just some issues that we know about. There are likely to be unknowable confounds that increase the amount of hidden error in a particular sample. As such, the types of data found in nomothetic approaches are faced with an inverse sample size problem: the noise-to-signal ratio increases exponentially with an increase in the size of the dataset. This is not to say that small data has a higher signal-to-noise ratio. But it does raise the problem that these various confounding factors in large datasets make finding a signal in amongst the noise increasingly difficult. As Taleb cogently puts it:

“This is the tragedy of big data: The more variables, the more correlations that can show significance. Falsity also grows faster than information; it is nonlinear (convex) with respect to data” [59].

## Evaluating Nomothetic Studies

We can use two of the issues above to evaluate nomothetic studies. First, the extent to which the experimental hypothesis is embedded in an existing theoretical framework. This relates to the hypothesised mechanism that causes the correlation that is presented. The second issue is the extent to which the study attempts to control for alternative hypotheses, particularly involving the historical relatedness of the observations. This relates to the strength of the correlation.

The interaction between these two issues lead to four types of study. First, there are studies that are motivated by prior theoretical and experimental work and are statistically rigorous. For example, the relationship between population size and morphological complexity (see above). This type of study can be valuable for testing hypotheses, generating hypotheses and acting as a catalyst for interdisciplinary work [14].

The second type of nomothetic study, which may also be valuable, includes studies which may not have been motivated by prior theories, but rigorously demonstrate that the hypothesised link is statistically sound. For example, Dediu and Ladd's study of genetic correlates of speakers of tonal languages demonstrated that their hypothesised link was significantly stronger than thousands of similar links. This type of study can be very useful for discovering new links that can motivate new avenues of research [13], especially when direct evidence is difficult to obtain. The link between tone and specific genes might have taken much longer to discover by small-scale studies. However, the statistical analysis does not directly support the hypothesised mechanism behind the link [14]. This must be done with methods other than nomothetic studies, such as experiments (e.g. [21]).

It is not always easy to judge whether the right controls are in place. Below we demonstrate a correlation between traffic accidents and linguistic diversity. This was not motivated by a prior theory, but it remains robust against controls for many factors. This type of study can be difficult to evaluate because the factors may be related in complex ways that are difficult to intuit about, or simply that the probability of a spurious correlation is increased in studies with large datasets.

The other two types of study may be detrimental. Those that are grounded in existing theories, but are poorly controlled risk missing hidden complexities which might challenge or develop the theory. For example, the study linking chocolate consumption and Nobel laureates (see above) was based on experimental findings on the cognitive benefits of chocolate. However, the statistical method was simply a linear correlation without any control variables. We find that the correlation does not remain significant when controlling for gross domestic product (GDP) and climate (see methods). More importantly, it is difficult to see what extra insight the this study provides over the controlled experiments that motivated it. This particular study has certainly gained public attention, but this might be dangerous if public opinion or policy is affected by poorly controlled studies.

Finally, studies that are not grounded in theory and are also poorly controlled can be misleading. It is difficult to distinguish these studies from ‘fishing’ for correlations from a large set of variables, then fitting a post hoc hypothesis to the strongest outcomes. As we demonstrate below, since cultural phenomena are subject to non-intuitive constraints, such as Galton's problem, it is relatively easy to produce evidence for a link between almost any two cultural variables that has the appearance of rigour. For example, we find that the per-capita consumption of chocolate also predicts the number of serial killers and rampage killers a country produces (see methods). There was no prior reason to think that this relationship would hold, apart from the likelihood of cultural traits being correlated. Despite this, it appears to support negative effects of chocolate, in opposition to the positive associations of the study above [9]. There is a danger that these methods could be exploited by researchers, politicians or the media to support particular agendas.

An example from economics highlights this danger. A well-cited study found a correlation between countries with a high ratio of national debt to GDP and countries with slow GDP growth [66]. The authors interpret this as economic growth being stifled by high debt. Although this goes against established theories [67], this interpretation has been widely cited in the media [68] and has been used in testimony before the US senate budget committee in order to support budget cuts [69]. However, the results have recently been shown to be an effect of poor statistical controls and the accidental exclusion of a cluster of related countries [68]. A more careful analysis revealed that countries with high debt to GDP ratios actually had positive growth [68]. Despite this radical change in implication, some commenters are already predicting that it will have little effect on policy, since the statistic was being used opportunistically to support claims for which theoretical arguments were more valid [70]. In this sense, correlational studies can be used as rhetorical devices with the appearance of rigour, but which actually have low explanatory power. Furthermore, damage caused by misleading studies may not be easy to fix.

The potential negative implications of nomothetic approaches can be addressed by applying more rigorous standards to statistical methods and increasing the awareness amongst researchers and the general public of the fragility of simple correlational studies. We hope to contribute to this awareness by demonstrating a chain of surprising links.

### Processes

**Figure 1:**
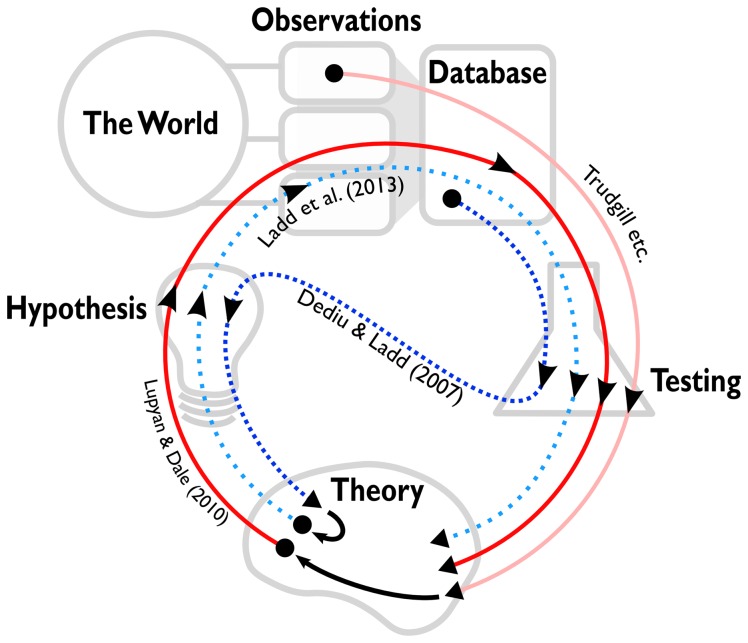
The scientific processes of different nomothetic studies. Observations are drawn from the world, either as idiographic studies or experiments. These observations can be compiled into large-scale cross-cultural databases. Scientific elements include theory, hypotheses and testing. Trajectories indicate the process of different studies. Processes start at a dot and continue in the direction indicated by the arrows. The ideal trajectory is the following: A theory generates a hypothesis. The hypothesis suggests data to collect, which is then tested. The results of the test feed back into the theory. Lupyan & Dale (2010) follow this trajectory, although they take their data from a large-scale cross-cultural database. Lupyan & Dale's theory was generated by previous testing of (small-scale) observations by Trudgill and others. The trajectory of Dediu & Ladd's study differs in two ways. First, the trajectory starts with large-scale cross-cultural data rather than small-scale observations. Secondly, the testing generates the hypothesis, which suggests a theory. However, Ladd et al. (2013) use this theory to motivate a hypothesis which is tested on experimental data. Since developing theories from small-scale observations takes time and effort, Dediu & Ladd's study has effectively jump-started the conventional scientific process.

Another way to think about the differences between nomothetic studies is by tracking the way they develop. The two useful types of study follow different processes (see figure 1). The ideal process of a study is for a theory to generate an experimental hypothesis, the hypothesis to suggest data to collect and a way to analyse or test them, and then the results of the analysis to feed back into a better understanding of the theory. The study on the relationship between population size and morphological complexity [1] follows this process trajectory, although it uses large-scale cross-cultural data. Of course, all theories have to start somewhere, and the theory that the study was based on was developed from small-scale idiographic data. This is an example of how a nomothetic approach can use large-scale data to test hypotheses suggested by small-scale studies.

The study of genetic correlates of linguistic tone [7] had a different trajectory. Here, there was no prior theory. Instead, a pattern in large-scale data suggested a hypothesis which was developed into a theory (see [71]). However, this theory went on to suggest an experimental hypothesis which was tested on small-scale experimental data [21]. This is an example of how a nomothetic study can use large-scale data to generate hypotheses that motivate small-scale, experimental studies.

The two approaches follow different approaches to science. The former fits with a hypothetico-deductivist approach, the latter fits with a more inductive approach to science (although the division between the two approaches is not always clear-cut) [72]. However, the small-scale study in the latter example also followed the more conventional scientific process. In this sense, since developing theories from small-scale observations takes time and effort, the latter nomothetic study jump-started the conventional scientific process.

## Results

### Chain of correlations

**Figure 2:**
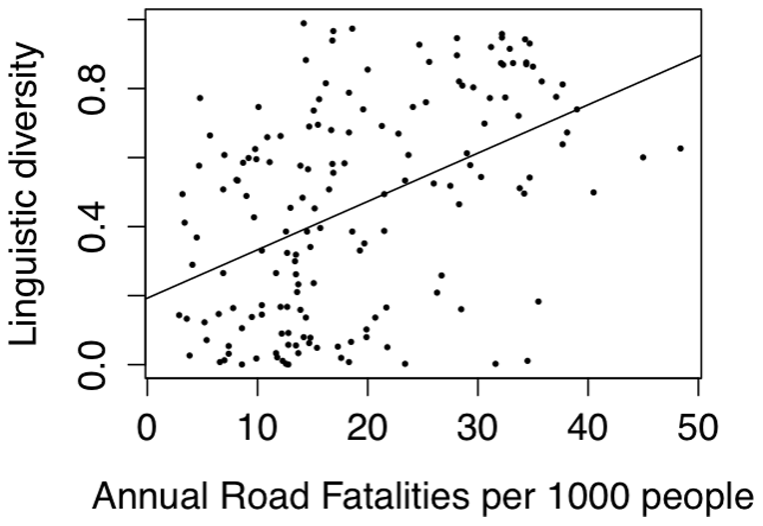
Linguistic diversity and traffic accidents. Countries' linguistic diversity (Greenberg diversity index) as a function of the annual traffic fatalities per 1000 people, with linear regression line.

If cultural traits are co-inherited, by descent or horizontal transmission, we should expect to find correlations between many cultural and demographic traits. For instance, the linguistic diversity of a country is correlated with the number of fatalities due to traffic accidents in that country, even controlling for country nominal GDP, per-capita GDP, population size, population density, length of road network, levels of migration, whether the country is inside or outside of Africa (a strong predictor of road fatalities), distance from the equator and absolute longitude (r = 0.45, F(97,10)  = 2.03, p = 0.003, see figure 2 and methods). This result is also robust to controlling for the geographic relationships between countries (r = 0.22, p = 0.000001, see methods).

**Figure 3:**
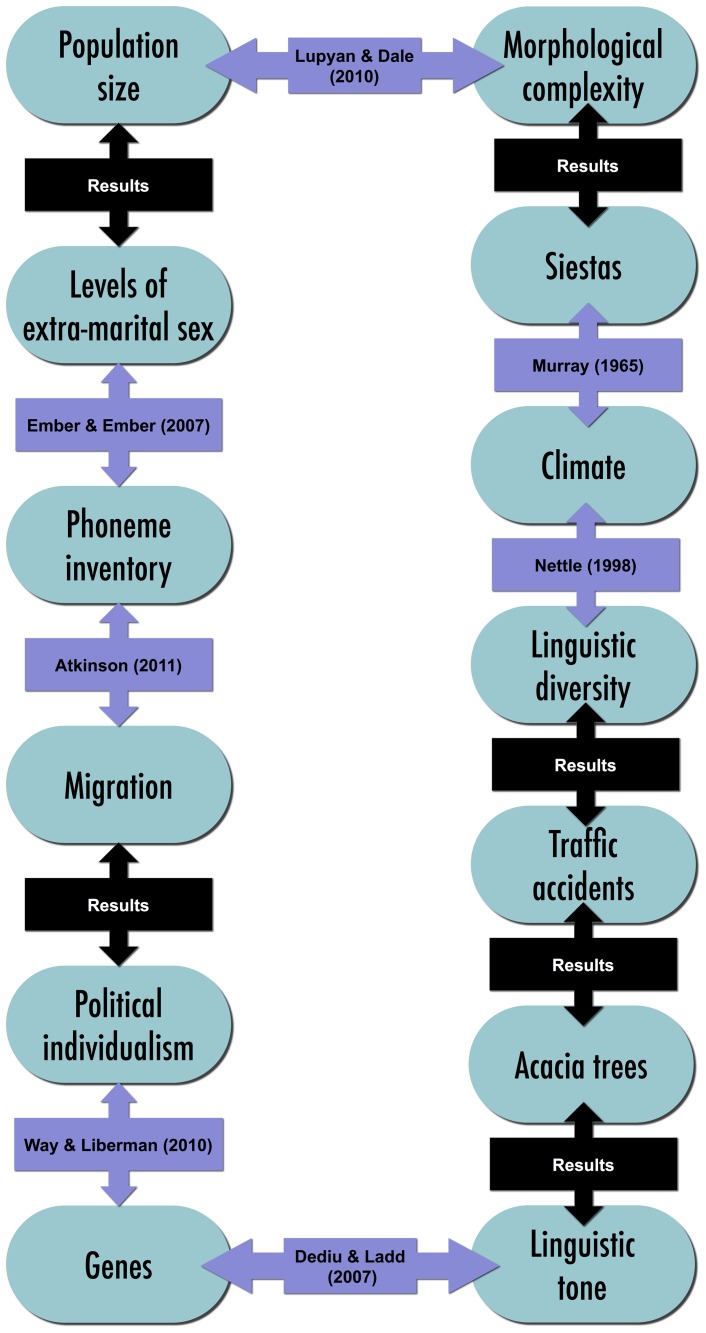
Chains of spurious correlations. Statistical links can be found between these cultural traits. Links from previous studies are labelled with the authors' names. The links from the results section of the current study are labelled ‘results’.

Furthermore, it is possible to demonstrate a chain of relationships between cultural variables (see figure 3): Linguistic diversity is linked with climate [73]. Climate affects the likelihood of cultural siestas [74]. Cultures that take siestas tend to have languages with less morphological complexity (t = 3.47, p = 0.001, see methods). Morphological complexity is linked with group size [1]. Group size is linked to the levels of extra-marital sex in a community (r = −0.54, p = 0.001, see methods). Levels of extra-marital sex have been linked to a language's phoneme inventory [6]. Phoneme inventories have been linked to patterns of migration [3]. Migration patterns are linked to the level of political collectivism in a culture (r = 0.42, p = 0.004, see methods). Collectivism is predicted by genetic factors [4], [5]. There are also genetic correlates of linguistic tone [7]. Tonal languages co-occur with acacia trees (t = 3.77, p = 0.0002, see methods). To bring the chain full-circle, the presence of *Acacia nilotica* also predicts a greater number of traffic accident fatalities, controlling for linguistic diversity, length of road network, GDP, distance from the equator, population size and population density (t = 3.26, p = 0.0014, see methods).

## Discussion

In the analyses above, we demonstrated a chain of correlations between cultural and demographic features. Some links are well motivated by prior hypotheses and statistically sound (e.g.[1], as discussed above). Others might not have had prior motivation, but are statistically sound and, in some cases, have gone on to be tested by experiments (e.g. [7], as discussed above).

In contrast, some of the studies fail the evaluation criteria discussed in the previous section. Some of the analyses are poorly controlled. For example, the link between acacia trees and tonal languages does not account for obvious environmental features such as temperature and altitude. However, some of the analyses appear statistically sound, but have no prior motivation and are not plausibly causally linked. For example, the link between traffic accidents and linguistic diversity controlled for many relevant factors. One could hypothesise that miscommunication between speakers of different languages could cause accidents, but it is more likely that a third variable such as the stability of the state explains both linguistic diversity and traffic safety. In this example, the confound is fairly obvious. However, as the number of variables involved increases, and the processes become more complex, it can become increasingly difficult to have intuitions that would lead to this resolution. Political stability might be an obvious control to include for a political scientist, but might not occur to a linguist. The kinds of aspects that nomothetic studies are being used for are typically on the border between two or more disciplines (Genetics and Linguistics [7]; Economics and linguistics [2]; Morphology, language change and demography [1]). Without a broad knowledge of these disciplines, or collaboration, this is exactly the kind of situation which might be difficult to intuit about.

The opposite problem – of knowing which variables to exclude from an analysis – may be equally difficult to answer. Since there is a chance that any cultural traits will be correlated, and since we actually demonstrate some above, there is an argument for including more control variables. For example, if a study investigates linguistic diversity, should it take the number of traffic accidents into account? Worse, since we demonstrate a chain of links, should a study of any of them control for all of the others? That is, if a study is interested in morphological complexity, should it take the collectivism of its speakers into account? For many methods, including more variables reduces statistical power and complicates the analysis. While intuition and theory play a role in knowing what to control for, in the next section, we suggest some practical solutions to these problems.

## Solutions

### Building better corpora

One of the most challenging issues to resolve is minimising the distance between those doing the data analysis and those researchers involved at other levels (e.g. field linguists). Part of the appeal of the nomothetic approach is the ease and cost-effectiveness in performing the analysis [14]. However, if the fundamental problems outlined in this paper are to be overcome, then there a few solutions we can apply to this distance problem which involve improving the data quality. First, we want to increase the resolution of each individual variable. So, to take the previous example of consonant inventory size, the aim should be to report all accounts and not select one on the basis of prior theoretical assumptions. Having more data per variable will increase the statistical power for nomothetic studies. Second, minimising distance can be achieved by using multiple and, ideally, independent datasets that work together to build up mutually supporting evidence for or against a particular hypothesis. Different datasets can take the shape of those derived from different large-scale studies (e.g. Phoible [75] and WALS for phoneme inventory counts [55]), idiographic accounts of individual case studies and experimental data.

Thirdly, databases such as the WALS indicate linguistic norms for populations, but may not capture the variation within and between individuals. One solution is for the primary data to be raw text or recordings of real interactions between individuals [76] and for population-level features, such as grammatical rules, to be derived directly from these. While collecting adequate amounts of data of this kind is more difficult, and while it is not free of biases, it offers a richer source of information.

Furthermore, databases should be collected and coded with specific questions in mind, otherwise there is a risk that correlations could emerge due to biases in the original motivation for the database. For example, the database that was used to demonstrate a link between future tense and economic behaviour was designed to identify similarities between European languages, which also happen to be culturally related and relatively wealthy [36].

### Model comparison

The correct null models to use when assessing cultural traits can be difficult to estimate, or unintuitive. As we shall demonstrate below, standard baselines of chance may not be conservative enough to eliminate spurious correlations. Rather than use random chance as a baseline, studies should compare competing hypotheses (as in [7]). Model comparison techniques allow researchers to test one model against another to see which better explains a particular distribution of data [77], [78]. So, whereas standard regression techniques are able to tell you the amount of deviance explained by a particular model, they do not provide information about whether you should have a preference for one model over another given a particular set of data. Model comparison techniques are therefore useful summaries of the available information and are better viewed as inductive-style approaches that should be complementary to the hypothetico-deductive and falisificationist approaches more typically associated with the scientific process [72]. Model comparison can also be used to test linear versus non-linear assumptions.

### Phylogenetic comparative methods

A simple, although conservative, test that controls for the relatedness of languages is to run the analysis within each language family (as in [1]). For example, the correlation between acacia trees and tonal languages is only significant for one language family, which is evidence against a causal relationship. However, more sophisticated methods are available. Studies of cultural traits have borrowed tools from biology to control for the non-independence of cultures [11]. Comparative methods include estimating the strength of a phylogenetic signal [49], [79] and estimating the correlation between variables while controlling for the relatedness of observations [80]–[82]. For example, in the analyses above we found that speakers who take siestas have grammars with less verbal morphology. While experiments show that daytime naps affect procedural memory [83], which has been linked to morphological processing [84], the predictions run in the opposite direction to the results. However, doing the same analysis, but accounting for the relatedness of languages using a phylogenetic tree [80], this correlation disappears entirely (r = 0.017, t = 0.13, p = 0.89, see methods). This highlights the very different implications that can come out of nomothetic studies when considering the independence of the observations.

While phylogenetic methods are relatively new and phylogenetic reconstruction (see below) is computationally expensive, software for phylogenetic comparative methods is freely available (e.g. packages for R, [85]–[88]) and do not require intense computing power. The more limiting factor for studies of linguistic features is a lack of standard, high-resolution phylogenetic trees.

Other phylogenetic techniques have been used to reconstruct likely trees of descent from cultural data (e.g. [89]–[91]). These may also be useful as further steps for determining whether links between cultural traits discovered by nomothetic studies are robust. For example, apparent universals in the distribution of linguistic structural features may actually be underpinned by lineage-specific trends [92].

### Causal graphs

Our analyses above suggests that cultural features are linked in complex ways, making it difficult to know what to control for in a specific study and potentially casting doubt on the value of nomothetic approaches. However, we see nomothetic studies as a useful tool for exploring complex adaptive systems. One change to the approach which could offer better resistance to the problems above would be to move away from trying to explain the variance in a single variable of interest towards analysing networks of interacting variables.

**Figure 4:**
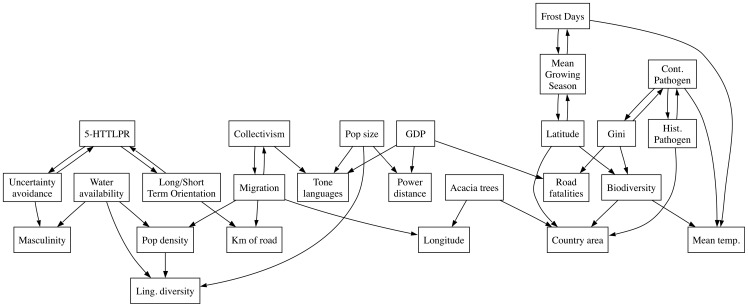
The most likely directed, acyclic graph of causal relationships between different variables in this study. Boxes represent variables and arrows represent suggested causal links going from a cause to an effect. See the methods section for details.

One method that could aid this type of analysis is the construction of causal graphs from large datasets [15]. While mediation analyses are often used to assess the causal relationship between a small number of variables [4], recent techniques are designed to handle high-dimensional data. We applied this technique to many of the variables in the study above. Figure 4 shows the most likely directed, acyclic graph that reflects the best fit to the relationships between the variables. We emphasise that this graph should be interpreted as a useful visualisation and as a hypothesis-generating exercise rather than representing proof of causation between variables.

Some interesting relationships emerge. First, some elements make intuitive sense, like the contemporary pathogen prevalence relying on the historical pathogen prevalence and the Gini coefficient (the balance between rich and poor within a country). Also, environmental variables like the number of frost days, mean growing season and mean temperature are linked.

More importantly, while the initial analysis above finds a direct correlation between linguistic diversity and road fatalities, even controlling for many factors, the causal graph analysis suggests that linguistic diversity and road fatalities are not causally linked. Instead, linguistic diversity is affected by demographic variables such as population size and density while road fatalities are affected by economic indicators such as GDP and the Gini coefficient. Similarly, the analysis suggests that tonal languages and the presence of acacia trees are not causally linked.

While the causal graph mainly provides evidence against some of the correlations above, it may also suggest interesting areas of further investigation. Interestingly, the causal graph suggests that collectivism is not directly linked with the genetic factors implicated by [4], but the relationship is mediated by (current) migration patterns. While speculative, it would be interesting to test the hypothesis that the distribution of genetic factors that are correlated with collectivism emerged by a process of selective migration (although see [93]). For example, the genotype that correlates with more collectivist countries is associated with a greater risk of depression under stress [29], so perhaps this gene came under selection in harsher climates. Indeed, we find some support for this idea, since adding environmental variables improves the fit of the model predicting the distribution of genotypes (compared to [4], see methods section). In this way, causal graph analyses may be a useful additional tool that can be used to explore relationships between complex adaptive variables such as cultural traits. Since the range of hypotheses suggested by inductive approaches can be very large, methods such as causal graphs can point to fruitful hypotheses to develop with more conventional approaches such as experiments.

## Conclusion

Due to increasingly accessible data and analysis methods, there has been a recent rise in studies that use large-scale cross-cultural databases to demonstrate correlations between cultural and demographic variables. While these studies may be useful for generating hypotheses and fostering interdisciplinary work, there are also problems which mean that they may have little explanatory power [14]. One of these problems is the relatedness of cultural groups and the correlated inheritance of cultural traits (Galton's problem). In this paper we illustrate the scale of the problem by demonstrating a chain of correlations between a diverse set of cultural traits. The probability of a spurious correlation between any two cultural traits is higher than is sometimes appreciated by researchers, the media and the general public.

We suggest four ways of addressing the problem of spurious correlations. First, better data will can reduce the likelihood of correlations generated by noise. Secondly, we suggest that null models should be derived from alternative hypotheses rather than random chance. Thirdly, we encourage the development of phylogenetic techniques that account for the relatedness of cultures. Finally, we suggest moving from a paradigm of trying to explain the link between two variables towards explaining networks of interacting variables.

Although the explanatory power of these studies is weak, the appearance of rigour in the correlational analysis gives the related hypotheses credibility. Given the potential implications on policy for some cultural phenomena, conclusions from nomothetic studies could have negative effects. Researchers and reviewers should be cautious when evaluating approaches which link variables that are related by descent.

## Materials and Methods

Here we describe the data and analyses used to demonstrate the spurious correlations between cultural variables discussed above.

### Linguistic diversity and traffic accidents

The first analysis compared the linguistic diversity of a country to the number of fatal traffic accidents. The analysis contained data from 117 countries. A multiple regression was carried out with the Greenberg diversity index [94] as the dependent variable and the following independent variables: Road fatalities per 100,000 inhabitants per year [95]; population size [94]; population density [96]; nominal GDP, per-capita GDP [97]; net migration rate [98]; absolute latitude; absolute longitude; whether the country was inside or outside of Africa (a strong predictor of road fatalities) and total length of road network [99]. The fit of the model is improved by adding road fatalities after entering all other variables (RSS  = 7.4781, F (106,1)  = 8.9, p = 0.0035). Model adjusted 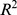 = 0.23, F(106,10)  = 4.56, p
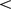
0.0001. Road fatalities coefficient  = 0.012, r = 0.45, data available in Supporting Information S1, file S1_01.csv).

### Siestas and morphological complexity

**Figure 5:**
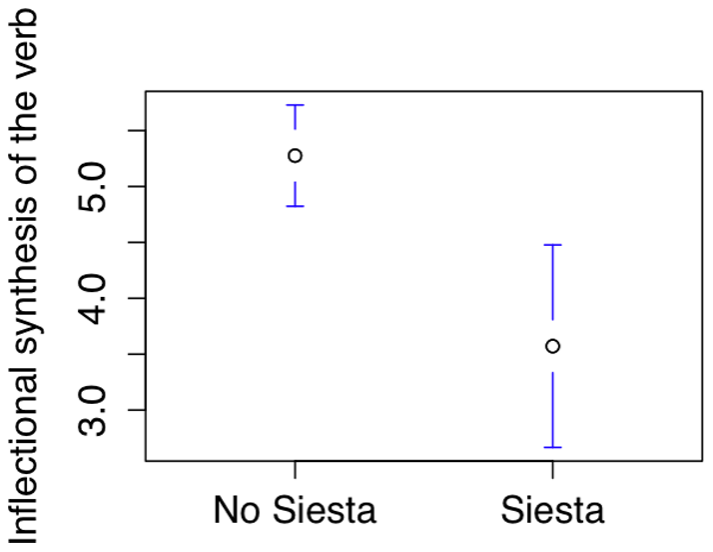
Relationship between siestas and number of grammatical categories a verb can take.

Countries with cultures of taking afternoon naps [100] are less morphologically complex, as measured by the mean number of grammatical categories a verb can take [101] (n = 137, t = 3.47, p = 0.001, see figure 5, Data available in Supporting Information S1, file S1_02.csv). Note that countries in Asia, Europe and South America take daytime naps. To test whether this is affected by Galton's problem, the language classifications for 127 languages were retrieved from the Ethnologue [102] and used to generate a phylogenetic tree (using H. Bibiko's AlgorithmTreeFromLabels program [103]). Without the phylogenetic tree, the correlation is significant (logit model: r = −0.36096, z = −2.755, p = 0.00586). To account for the phylogenetic tree, a generalised estimating equations test was run with binomial response distribution [80], [85] (comparison suggested by chapter 7.7 of [88]). In this case, the correlation disappears (r = 0.017, t = 0.13, p = 0.9, dfP  = 63.2, estimated scale parameter  = 1.17, data available in Supporting Information S1, file S1_02b.zip).

### Extramarital sex and population size

**Figure 6:**
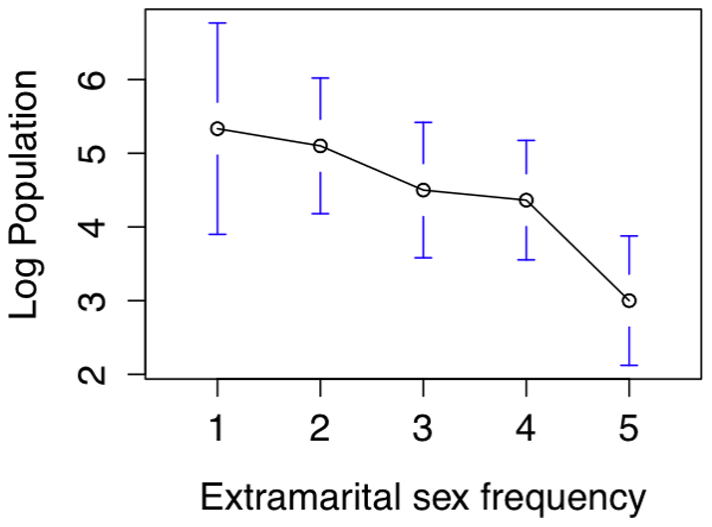
Relationship between population size and frequency of extramarital sex in a society.

Extramarital sex is correlated with population size (r = −0.54, df = 31, p = 0.001), data from [104]. A regression using population size as the dependent variable and independent variables including population density and four measures of sexual attitudes showed that extramarital sex frequency was the best predictor after population density (see figure 6 and table 1, adjusted 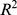 = 0.38, F (26,5)  = 4.84, p = 0.003, Data available in Supporting Information S1, file S1_03.csv.).

**Table 1:** Population size and extramarital sex.

	Coefficient	Std. Error	t value	Pr(>|t|)	
(Intercept)	7.40	1.94	3.82	0.01	*
Population density	0.32	0.11	2.85	0.01	*
Premarital sex frequency	−0.39	0.21	−1.90	0.07	
Premarital sex deterrence	−0.22	0.34	−0.65	0.52	
Extramarital sex frequency	−0.38	0.17	−2.21	0.04	
Extramarital sex deterrence	−0.31	0.30	−1.05	0.31	

Results of a regression using population size as the dependent variable and independent variables including population density and four measures of patterns of and attitudes to extramarital sex.

### Migration and Collectivism

**Figure 7:**
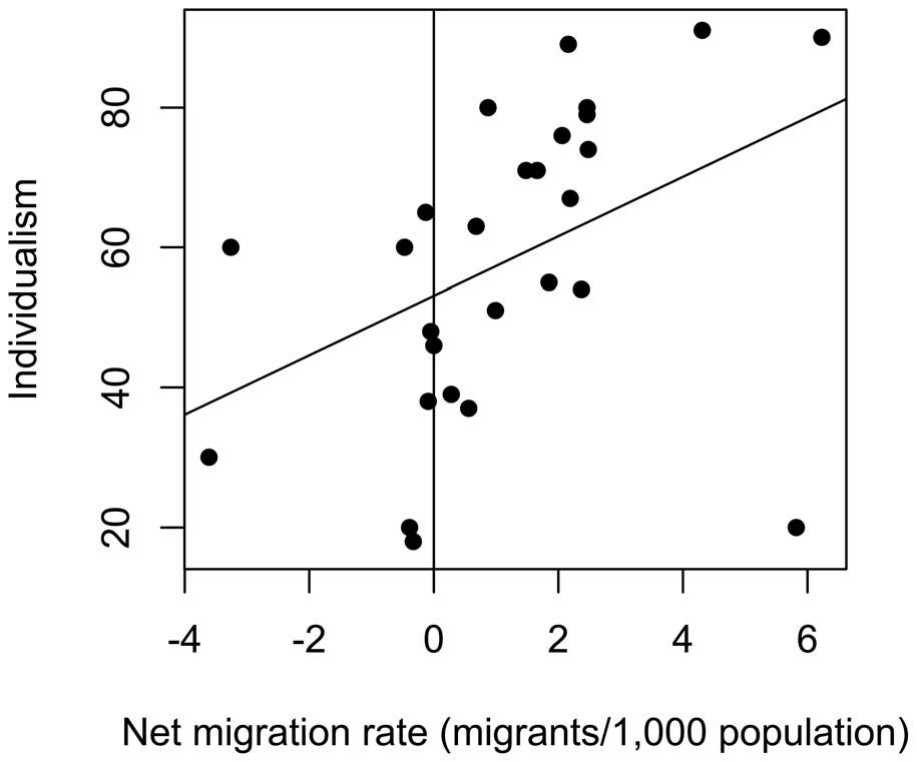
Individualism-Collectivism as a function of migration. Data shown for 28 countries with linear regression line. Large values on the y-axis indicate greater individualism.

Cultural values of collectivism are related to the prevalence of an allele of the serotonin transporter functional polymorphism 5-HTTLPR [4]. The original study used a linear regression with a measure of a country's collectivism as the dependent variable and independent variables including the prevalence of the 5-HTTLPR short-short allele, GDP, Gini coefficient and measures of pathogen prevalence. We replicated exactly the original finding that prevalence of the short-short allele is a significant predictor of collectivism (coefficient  = −0.85, t = −2.94, p = 0.0079). Adding contemporary migration levels [98] shows that migration levels are a significant predictor (n = 28, r = 0.42, see figure 7 and table 2) and improves the fit of the model (RSS difference  = 1334, F(22,1)  = 10.3, p = 0.004, adjusted 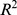 = 0.73, data is available in Supporting Information S1, file S1_04.csv). Higher levels of collectivism (lower levels of individualism) correlate with lower migration rates.

**Table 2:** Genes, collectivism and migration.

	Estimate	Std. Error	t value	Pr(>|t|)	
(Intercept)	119.0229	30.6819	3.88	0.0009	
5-HTTLPR short-short allele prevalence	−1.1268	0.2478	−4.55	0.0002	*
Gini coefficient	−0.5244	0.2977	−1.76	0.0935	
Contemporary pathogen prevalence	0.3800	0.8025	0.47	0.6409	
Historical pathogen prevalence	−7.7133	6.3512	−1.21	0.2387	
GDP	−0.0002	0.0003	−0.71	0.4855	
Net migration	5.0025	1.5565	3.21	0.0044	*

A linear regression with levels of collectivism in a country as the dependent variable and independent variables including the prevalence of an allele of the serotonin transporter functional polymorphism 5-HTTLPR, Gross Domestic Product, Gini coefficient, measures of pathogen prevalence and contemporary migration levels.

Furthermore, we find that adding environmental variables (biodiversity [105], mean minimum annual temperature [106] and mean growing season [105] improves the fit of the model on top of the contribution from migration (RSS difference  = 1318.8, F(16,4)  = 4.1745,p = 0.017, adjusted 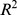 = 0.83, see table 3).

**Table 3:** Genes, collectivism, migration and ecology.

	Estimate	Std. Error	t value	Pr(>|t|)	
(Intercept)	121.6578	25.6328	4.75	0.0002	*
5-HTTLPR short-short allele prevalence	−1.0900	0.2012	−5.42	0.0001	*
Gini coefficient	−0.4310	0.2694	−1.60	0.1292	
Contemporary pathogen prevalence	−0.6594	0.6995	−0.94	0.3599	
Historical pathogen prevalence	−5.5039	5.9007	−0.93	0.3648	
GDP	−0.0002	0.0003	−0.74	0.4717	
Net migration	4.1561	1.3598	3.06	0.0075	*
Biodiversity	0.1925	0.0718	2.68	0.0163	*
Minimum average temperature	2.7335	0.8841	3.09	0.0070	*
Mean growing season_calc	2.9013	1.3535	2.14	0.0478	
Minimum average temperature:					
Mean growing season_calc	−0.3352	0.1049	−3.20	0.0056	*

### Tone and Acacia Trees

**Figure 8:**
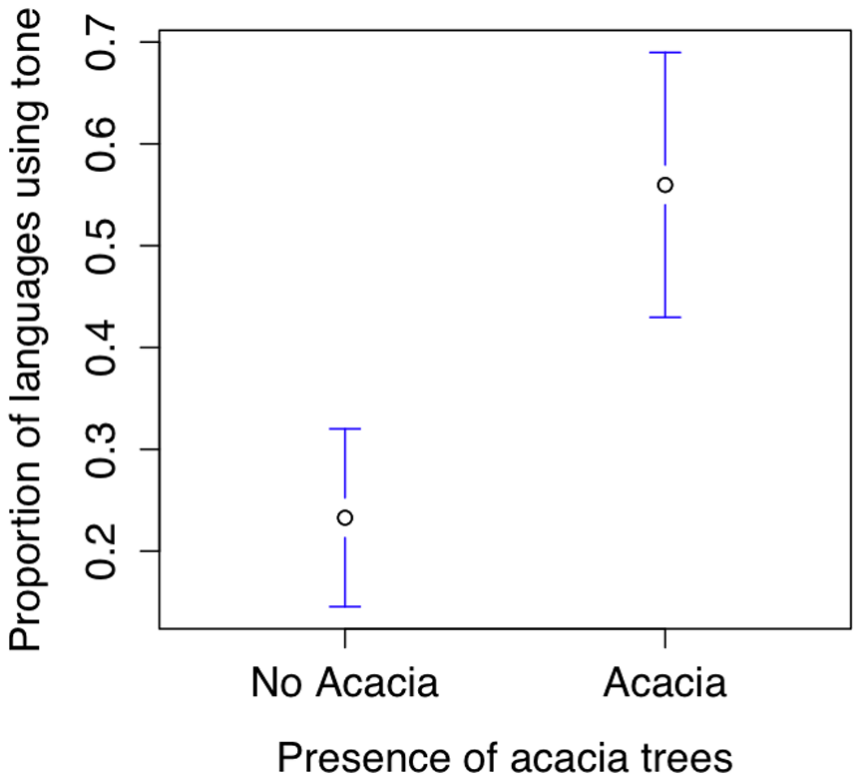
The proportion of tone languages within a country as a function of the presence of *Acacia nilotica*.

Countries in which the acacia tree *Acacia nilotica* grows [107] were compared with countries which include tone languages (languages that use “pitch patterns to distinguish individual words or the grammatical forms of words”, [108]). Acacia trees and tone languages (simple or complex) co-occur with a probability greater than chance (617 languages in 114 countries, 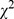 with Yates' continuity correction  = 47.1, df  = 1, p
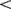
0.0001, see also [109], data available in Supporting Information S1, file S1_05.csv). The proportion of tonal (vs. non-tonal) languages in a country is significantly higher if that country has acacia trees (mean proportion of languages using tone in countries with acacia trees  = 55.9% (41 countries), without acacia trees  = 23.3% (73 countries), t = 4.2, df = 76, p = 0.00007, see figure 8). The proportion of languages with linguistic tone in a country predicts the presence of acacia trees, even when controlling for latitude (linear model, tone coefficient  = 0.39, t = 3.77, p = 0.0002).

We can run an analysis of the relationship between tone and acacia trees within each language family. Enough data and variance was available for 8 language families (see table 4). The relationship was only significant for languages from the Niger-Congo family.

**Table 4:** Tone and acacia trees by language family family.

Family	Observations	t	p
Afro-Asiatic	29	−1.11	0.29
Austro-Asiatic	16	0.66	0.54
Austronesian	42	−1.73	0.17
Indo-European	30	0.27	0.81
Niger-Congo	64	4.99	0.000006
Nilo-Saharan	26	1.37	0.19
Sino-Tibetan	25	1.98	0.06
Trans-New Guinea	19	0.88	0.40

Results of t-tests for the relationship between linguistic tone and the presence of acacia trees within different language families. Columns indicate the language family, the number of languages used as observations in the test, the t-test statistic of the difference between tonal and non-tonal languages in terms of the presence of acacia trees and the probability value associated with that t-value.

### Acacia Trees and traffic accidents

**Figure 9:**
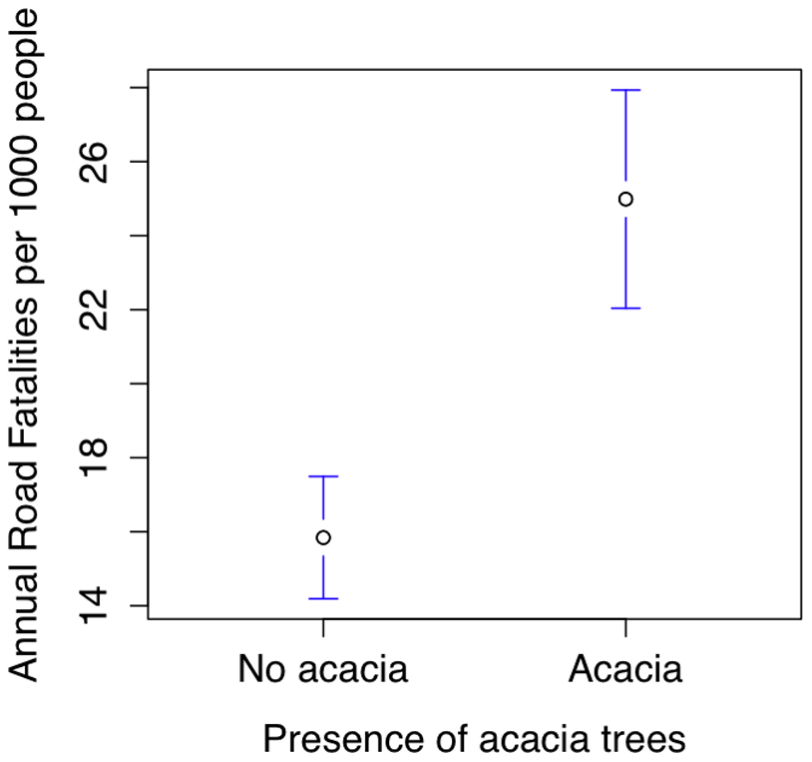
The mean number of annual road fatalities per 100,000 people within a country as a function of the presence of *Acacia nilotica*.

Countries in which the acacia tree *Acacia nilotica* grows [107] have higher incidences of road fatality [95] (see figure 9, mean road fatalities per 100,000 inhabitants per year in countries without acacia trees  = 15.84, mean in countries with acacia trees  = 24.98654, df = 85, p = 0.0000006). A linear regression predicting the levels of road fatalities using presence of acacia trees, km of road [99], greenberg diversity index [94], nominal GDP [97], absolute latitude, population size [94] and population density [96] (see table 5), shows that the presence of acacia trees is a significant predictor (adjusted 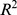 = 43.1%, data available in Supporting Information S1, file S1_6.csv).

**Table 5:** Traffic accidents and acacia trees.

	Estimate	Std. Error	t value	Pr(>|t|)	
(Intercept)	18.5610	1.8427	10.07	<0.0001	*
Presence of acacia trees	4.9211	1.5106	3.26	0.0014	*
Length of road network	−0.0067	0.0038	−1.77	0.0790	
Greenberg diversity index	10.3095	2.1952	4.70	<0.0001	*
Nominal GDP	−0.0000	0.0000	−1.30	0.1949	
Distance from equator	−0.1524	0.0414	−3.68	0.0003	*
Population size	−0.0000	0.0000	−1.09	0.2781	
Population density	−0.0038	0.0010	−3.65	0.0004	*

A linear regression predicting the levels of road fatalities using presence of acacia trees, km of road, greenberg diversity index, nominal GDP, absolute latitude, population size and population density.

To test the geographic relatedness of countries, the distance between each country in the sample was calculated (great circle distance from the center of each country) to produce a geographic distance matrix. Similar distance matrices were made for the GDI and road fatalities variables (absolute difference between countries). A Mantel test was used to calculate the probability of a correlation between GDI and road fatalities (r = 0.22, p = 0.000001, one million permutations). This remained significant when controlling for geographic distance with a partial Mantel test (r = 0.22, p = 0.000001, although see [48] for problems with Mantel tests).

### Chocolate consumption and serial killers

We take five variables from Wikipedia: the number of Nobel prizes awarded by country of recipient (and the population of that country) [110]; The nominal gross domestic product (GDP) per capita [111]; the number of road fatalities per 10,000 population [112]; the number of serial killers since 1900 [113] and the number of rampage killers since 1900 [114]. The average annual temperature was obtained [105]. Data on the average IQ of the populations of different countries were obtained from [115]. The collected data is available in the supporting materials (Supporting Information S1, file S1_07.csv).

**Figure 10:**
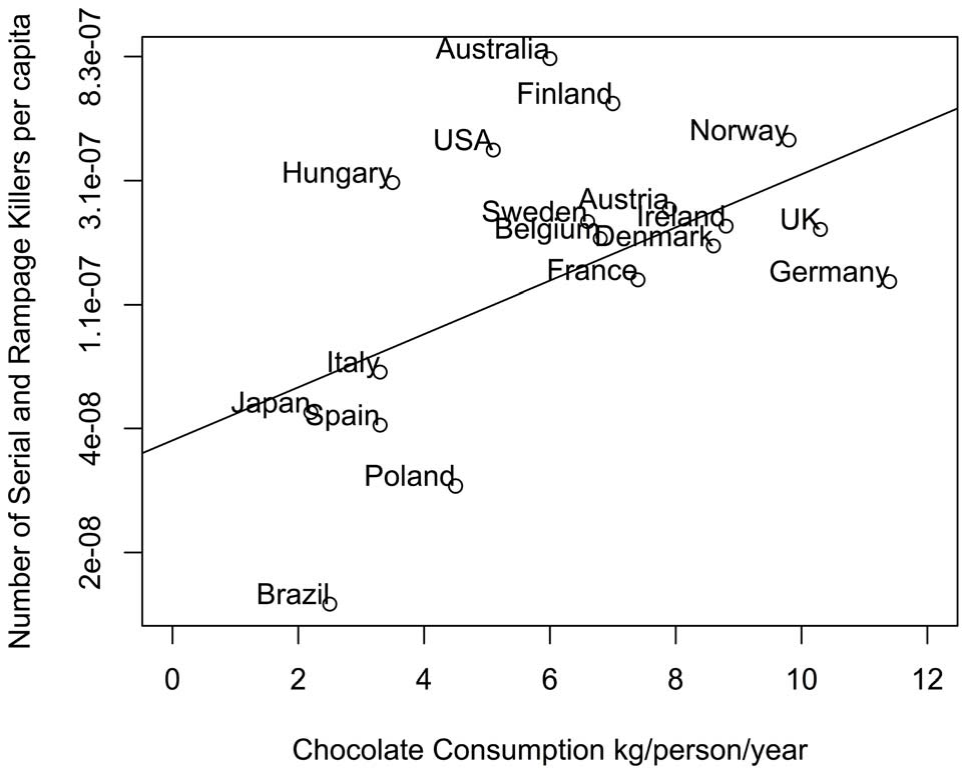
Chocolate consumption per capita (kg) as a function of the log number of serial and rampage killers per capita since 1900.

We replicated the finding from [9] that chocolate consumption per capita correlates with the number of Nobel laureates per captia (r = 0.73, p = 0.00007). However, a linear regression controlling for per-capita GDP and mean temperature found that chocolate consumption was not a significant predictor of the number of Nobel laureates (F(1,19)  = 3.6, p = 0.07). Countries with higher GDP and lower mean temperatures correlate with higher Nobel laureates per capita (r = 0.7, −0.6, p = 0.0002,0.0016). Furthermore, the average IQ of a country did not correlate with chocolate consumption (r = 0.27, p = 0.21). Additionally, for 18 countries where data was available, the level of chocolate consumption per capita is significantly correlated with the (log) number of serial killers and rampage killers per capita (r = 0.52, p = 0.02, see figure 10). We assume that there is no causal link here. Also, we found that the number of road fatalities per 100,000 inhabitants per year correlates with the number of Nobel Laureates (r = −0.55, p = 0.0066), which we also assume has no causal link.

### Causal graphs

The data from the studies above were aggregated over countries and combined into a single dataset. We used the PC algorithm [116] as implemented in the R package pcalg [117] to compute the most likely directed acyclic graph of relationships between variables. The algorithm has a parameter that determines the threshold at which links should be included. The results come from using the smallest threshold that included all the variables in a single connected component. We note that the exact causal links that are selected are sensitive to this parameter and to different subsets of the data. Therefore, we suggest that this method is only an exploratory tool rather than a formal proof of relationships. We look forwards with anticipation to the development of this tool.

## Supporting Information

Supporting Information S1 **Contains: S1_01.csv**: Data on road fatalities, linguistic diversity and demographic variables for countries. **S1_02.csv:** Data on Siestas and morphological complexity. **S1_02b/AlgTree_ASJP_Ethno.nwk**: Phylogenetic tree of languages. **S1_02b/s.csv**: Data on Siestas and morphological complexity. **S1_02b/Siesta_PhyloLogit.r** R script for running phylogenetic generalised estimating equations test. **S1_03.csv**: Data on population size and extramarital sex frequency. **S1_04.csv**: Data on genetic correlates of collectivism and migration. **S1_05.csv**: Data on Tone langauges and Acacia trees. **S1_6.csv:** Data on Acacia tree and traffic accidents. **S1_07.csv**: Data on Chocolate consumption and serial killers.
(ZIP)
